# Theoretical study of the synergic relationships between the design parameters in energy-saving building design

**DOI:** 10.1038/s41598-024-53735-4

**Published:** 2024-02-22

**Authors:** Hai ’E. Huo, YanHong Ji, YuanYuan Qin, ChaoZheng Chen, Ting Yuan

**Affiliations:** 1 School of Architecture and Civil Engineering, Xihua University, Chengdu, 610039 China; 2 PengXi County Construction Engineering Safety and Quality Supervision and Management Station, Suining, 629000 China; 3 China 19T’’ Metallurgical Group Corporation Limited, Chengdu, 610039 China

**Keywords:** Synergic relationship, Discomfort indoor degree hours, Energy-saving rate, Thermophysical properties, Improved genetic algorithm (IGA), Energy science and technology, Engineering, Materials science

## Abstract

With the rapid development of the economy, people have increasingly higher requirements for the comfort of living spaces, and the result is the sharp increase in building energy consumption. Several design parameters influence living space comfort and building energy efficiency. Since the same design standard can include different design parameter combinations, synergic relationships may exist between these criteria for one case. Identifying these synergic relationships requires an inverse problem approach. This paper established a model by combining an improved genetic algorithm (IGA) and numerical calculation to determine the synergic design parameter relationships (e.g. the thermophysical building material properties and energy-saving factors). For ${{\text{I}}}_{{\text{sum}}}= {0}$, the shading coefficient significantly influenced the linear function between the thermal conductivity and insulation thickness. In this case, the insulation thickness was exponentially related to the shading coefficient, while the thermal conductivity of the insulation material significantly impacted the synergic relationship. For ${\text{ESR}}{=65}\%$, the insulation thickness was a segmented function of the shading coefficient. The results verified that the proposed model was efficient and reliable for identifying the synergic relationships between energy-saving parameters. In engineering applications, designers can select the optimal design parameter combination based on the relationship curve between the parameters in this paper according to the local market conditions and specific design requirements.

## Introduction

Due to rapid economic development in China, individuals are becoming more conscious of the comfort of their homes and the energy-saving qualities of buildings with energy-efficient designs^1–4^. Selecting the proper enclosure structures and energy-saving measures is vital for ensuring indoor thermal comfort and building energy efficiency. The energy-saving technologies commonly used in China include external roof and wall insulation, natural ventilation, and shading^5^. The relevant parameters include thermal conductivity and thickness of the insulation materials, shading co-efficient values, and ventilation times. Furthermore, the thermophysical criteria of the building envelope include thermal resistance and inertia, the heat transfer coefficient, and specific heat capacity. These parameters significantly influence the indoor thermal environment and building energy consumption^6–10^. Therefore, selecting suitable parameters is crucial for ensuring indoor comfort and reducing building energy consumption during energy-saving design. Synergic relationships may exist between these parameters.

Initial design decisions significantly affect thermal building performance^11,12^, focusing on selecting appropriate design parameters, which can be challenging to change at a later stage^13^.

Various studies have examined parameter optimization in building design. Target optimization can reduce building energy consumption, decrease lifecycle costs, lower CO_2_ emissions, improve indoor air quality, and increase indoor comfort. Moussaoui F. et al.^14^ used the performance-based strategy to develop a technique for analyzing residential building energy performance in the Algerian context. Genetic algorithms (GAs) and the analytic hierarchy process (AHP) were combined to determine the selected PI weights, which involves single-objective decision matrix optimization. Zhang J. et al.^12^ highlighted the significant potential of utilizing parametric optimization during the early design phase of green residential buildings to increase performance. The results indicated that optimizing the building envelope and spatial form parameters during the design phase can reduce the energy consumption of residential buildings. Xue Q. et al.^15^ proposed multi-objective, simulation-based optimization to reduce CO_2_ emissions and the lifecycle cost of buildings. N. Delgar, B. et al.^16^ proposed a single-objective and multi-objective particle swarm optimization (MOPSO) algorithm combined with building energy simulation software (EnergyPlus) to find a set of non-dominant solutions to improve the energy efficiency performance of buildings. Sim M. et al.^17^ proposed a multi-objective, particle swarm model to optimize lifecycle cost and energy savings according to PV capacity. Multi-objective building optimization has recently attracted increasing research attention^18–21^. Moreover, combining response surface approximation (RSA) models, including support vector machines (SVM) and artificial neural networks (ANN) with GA, represent the simulation-based optimization methods used in the building sector^22–26^. GA is highly efficient in solving optimization challenges during the building design stage. To sum up, the relevant case studies are shown in Table 1.

**Table 1:** The relevant case studies.

Author	Method	Research objective	Optimization result
Moussaoui F. et al.^14^	Genetic algorithms (GAs) and the analytic hierarchy process (AHP)	Develop an energy performance index for residential buildings	Influence of the climate aspect
Zhang J. et al.^12^	Genetic algorithm (GA)	Rreduce the energy consumption of residential buildings	Combination of spatial form and building envelope
Xue Q. et al.^15^	Non-dominated sorting genetic algorithm NSGA-II	Minimize both life cycle cost and CO_2_ emissions of buildings	Optimal combination of insulation thickness, window type, window-to-wall ratio, overhang depth and building orientation
N.Delgarm, B. et al.^16^	Multi-objective particle swarm optimization (MOPSO)	Enhance the building energy performance	Effect of the building orientation, the shading overhang specifications, the window size, and the glazing and the wall material properties
Sim M. et al.^17^	Multi-objective particle swarm optimization (MOPSO)	Maximize lifecycle cost and energy savings	Optimal combination of solar thermal (ST) and ground source heat pump (GSHP)
Shao T^19^	Meighted sum method	Find the best combination of design parameters	The best combination of building orientation, insulating layer thickness, window width & type, and indoor design temperature
Ciardiello A^20^	Genetic algorithm (GA)	Reduce the energy usage and emissions of buildings	Optimization of shape and envelopes variables

Mono- and multi-objective optimization in the building sector has attracted considerable research attention. Previous studies have focused more on optimal design strategy combinations involving "point-to-point" case studies, with less attention to the synergic relationships between the building design parameters and energy-saving technology factors. The authors' previous research^27^ combined improved genetic algorithms (IGAs) with numerical calculations to investigate the synergic thickness and thermal conductivity relationship of thermal insulation materials under different constraints for a south-facing wall. Chengdu belongs to the hot summer and cold winter region, with both summer cooling and winter heating demands. Due to its harsh climate conditions, the task of energy-saving is more challenging. Therefore, in this article, a single building in Chengdu is selected as the research object, and IGA is used as the search engine to explore the possible synergistic relationship between energy-saving measures and thermal performance parameters of building materials, with indoor thermal comfort and ESR as constraint objectives. The purpose of this study is to provide a basis and reference for engineering applications.

## Methods

### Building model description

#### Physical model

**Figure 1:**
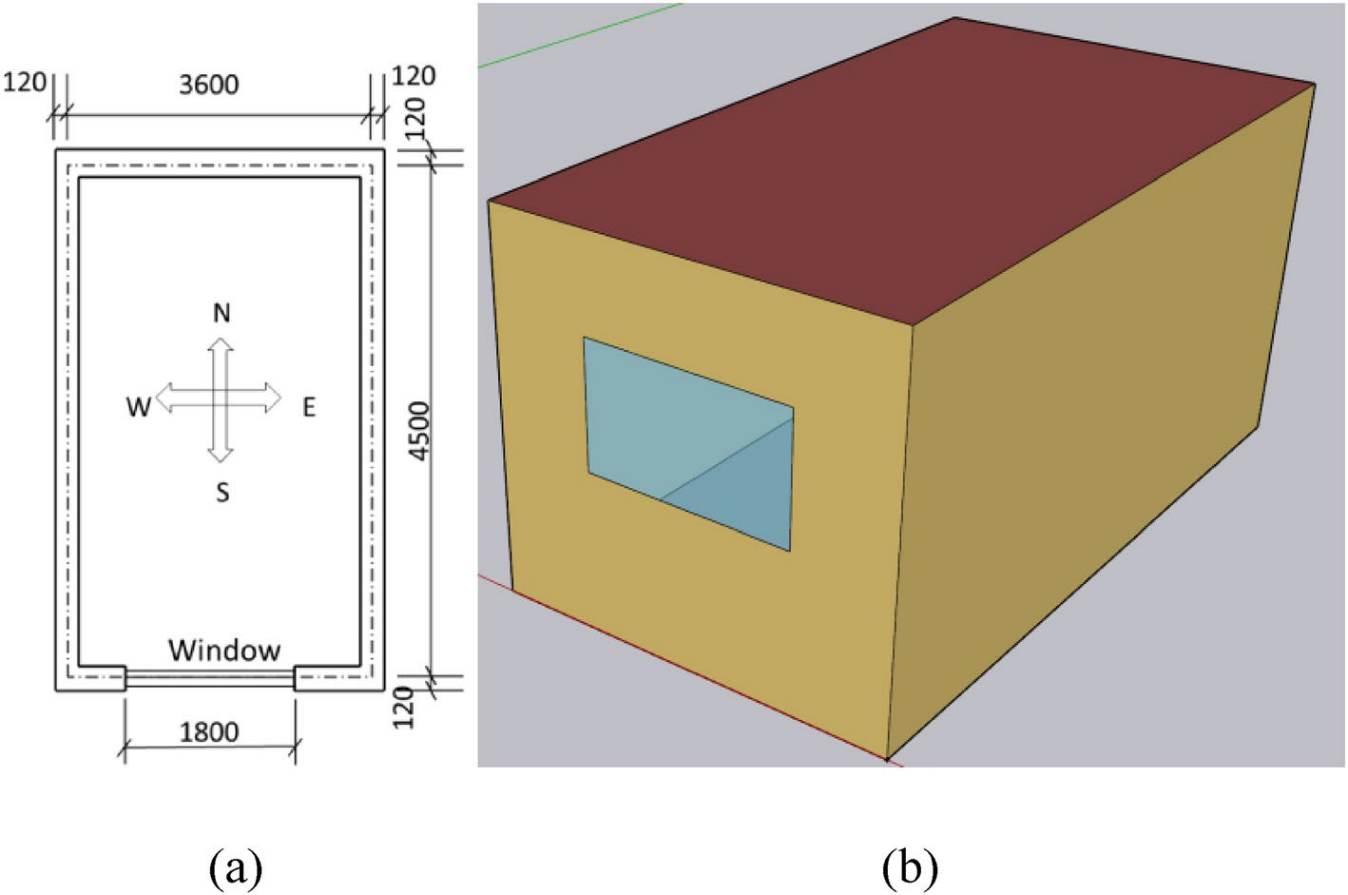
A schematic diagram of the simulated room. (**a**) A planar graph. (**b**) A stereoscopic diagram.

This paper selected a typical single-zone building in Chengdu, a representative city in the Chinese cold winter and hot summer zone, for investigation. Figure 1 shows its geometry, while Table 2 lists the building information.

**Table 2:** The building data.

Building construction	Description
Window	Single-glazing (3 mm) (plastic-steel window): 1.8 m (w) × l.5 m (h) U_win_ = 5.9 W/(m^2^⋅K)⋅$\, {\upvarepsilon }_{win}={0.94}$ ${\uptau }_{win}={0.82}$ α_win_ = 0.12^28^
Geometry	4.5 m (D) × 3.6 m (W) × 3.0 m (H) Four external walls, one roof, and one floor
Floor	Upper plaster (20 mm) + reinforced concrete (200 mm) + lower plaster (20 mm)
Roof	External plaster (20 mm) + reinforced concrete (200 mm) + internal plaster (20 mm)

The following assumptions were made for analytical convenience: (1) Because the height (length) and width of the building were more than 10 times its thickness, the wall, roof, and floor heat transfer were regarded as one-dimensional^29^. (2) Considering the symmetry, the floor was insulated at the matching center line. (3) Solar and internal radiation were evenly dispersed on the surface of the enclosure structure. (4) 70% of the solar radiation entering the room was absorbed by the floor, while the remaining 30% was distributed equally to the inner surface of other envelopes by area^30^. (5) Minimal inter-layer, thermal contact resistance was evident. (6) The inside air was fully blended. (7) Each surface was regarded as diffuse.

#### Mathematical model

**Figure 2:**
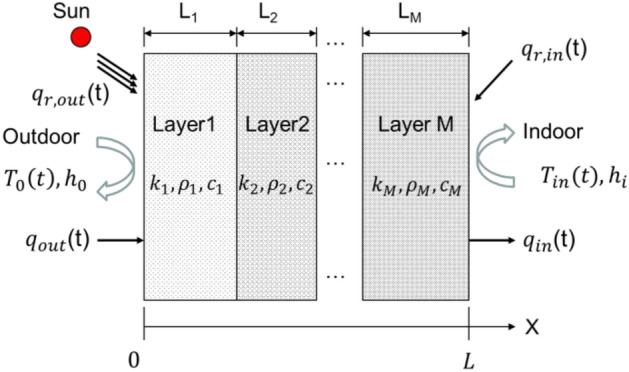
A heat transfer model of the multilayer composite structure.

Figure 2 shows a schematic of the mathematical model for the composite structure. The exterior envelope surface (except the floor) was exposed to solar radiation and the local temperature of the environment. The interior surface was exposed to room air, while the radiation between the inner surfaces was considered. Table 3 presents the thermal characteristics of the construction material used in this paper.

**Table 3:** The thermophysical characteristics of the construction material^31^.

Material	*λ*(W/(m^2^⋅K))	$\rho$(kg/m^3^)	${c}_{p}$(J/kg ∙ K)
Brick	0.81	1800	1050
Air	–	1.2	1013
Cement plaster	0.93	1800	1050
Window	5.9	2500	840
Reinforced concrete	1.74	2500	920

##### Mathematical description of the exterior wall, roof, and floor.

*Governing equation* Based on the above assumptions, the following equation is used for the one-dimensional transient heat conduction of the multilayered structure:

1 $$ \rho_{j} c_{pj} \frac{{\partial T_{j} }}{\partial t} = \lambda_{{\text{j}}} \frac{{\partial^{2} T_{j} }}{{\partial x^{2} }}{ }j = 1,2, \ldots ,{\text{M}} $$

where $x$ represents the space coordinate, $t$ denotes the time, ${T}_{j}$ signifies the temperature, and ${\rho }_{j}$,${c}_{pj}$, and λ_j_ refer to the $jth$ layer density, constant-pressure volumetric-specific heat, and thermal conductivity, respectively.

*Boundary conditions* The Eq. (1) boundary conditions were:

Exterior

2 $$   {\text{q}}_{{{\text{r,out}}}}  + {\text{ h}}_{{{\text{out}}}} \left( {{\text{T}}_{{{\text{out}}}}  - {\text{ T}}_{{{\text{x }} = 0}} } \right) = \left. { - \lambda _{1} \frac{{\partial {\text{T}}}}{{\partial {\text{x}}}}} \right|_{{{\text{x }} = 0}} ,\;\left( {{\text{for the external wall and roof}}} \right) $$

3 $$ \left. { - \lambda _{2} \frac{{\partial {\text{T}}}}{{\partial {\text{x}}}}} \right|_{{{\text{x }} = {\text{ }}\frac{{\text{L}}}{2}}}  = 0{\text{, (for the center of the floor)}} $$

Interior

4 $$ {\text{q}}_{{{\text{r,in}}}}  + {\text{ h}}_{{{\text{in}}}} \left( {{\text{T}}_{{{\text{in}}}}  - {\text{T}}_{{{\text{x }} = {\text{ L}}}} } \right) = \left. {\lambda _{{\text{M}}} \frac{{\partial {\text{T}}}}{{\partial {\text{x}}}}} \right|_{{{\text{x }} = {\text{ L}}}} , $$

where ${h}_{out}$ and ${h}_{in}$ represented the coefficients of the convective heat transfer at the exterior and interior composed structure surfaces, respectively, ${T}_{out}$ and ${T}_{in}$ denote the exterior and interior air temperatures, respectively, ${q}_{r,out}$ is the net exterior thermal radiation heat flux, primarily from sunshine, ${q}_{r,in}$ is the interior net thermal radiation heat flux, primarily from the exchange of radiation between the interior envelope surfaces and window heat transfer.

*Initial conditions*

5 $$ \left. {{\text{T(x,t)}}} \right|_{{{\text{t }} = {\text{ 0}}}}  = {\text{T}}_{{{\text{init}}}} , $$

where ${T}_{init}$ is the initial temperature.

##### Mathematical description of the exterior window

The following equation is used to calculate the window heat transfer:

6 $$ \rho _{{{\text{win}}}} {\text{c}}_{{{\text{pwin}}}} {\text{L}}_{{{\text{win}}}} \frac{{{\text{dT}}_{{{\text{win}}}} }}{{{\text{dt}}}} = {\text{q}}_{{{\text{r,out,win}}}}  + {\text{h}}_{{{\text{out,win}}}} \left( {{\text{T}}_{{{\text{out}}}}  - {\text{T}}_{{{\text{win}}}} } \right) + {\text{h}}_{{{\text{in,win}}}} \left( {{\text{T}}_{{{\text{in}}}}  - {\text{T}}_{{{\text{win}}}} } \right) + {\text{q}}_{{{\text{r,in,win}}}} , $$

where ρ_win_, ${\text{c}}_{\text{pwin}}$, and ${\text{L}}_{\text{win}}$ represent the window glass density, specific heat, and thickness, respectively, ${\text{h}}_{\text{out,win}}$ and ${\text{h}}_{\text{in,win}}$ denote the coefficients of the convective heat transfer between the exterior and interior window glass surfaces, respectively, ${\text{q}}_{\text{r,out,win}}$ and ${\text{q}}_{\text{r,in,win}}$ signify the radiant heat fluxes absorbed by the outer and the inner surfaces of the window glass, respectively, and ${\text{T}}_{\text{win}}$ is the temperature of the window glass. 

##### Mathematical description of the indoor air

Since this study did not consider the internal heat source, the indoor air energy conservation equation can be expressed as:

7 $$ \rho _{{\text{a}}} {\text{c}}_{{{\text{pa}}}} {\text{V}}_{{\text{R}}} \frac{{{\text{dT}}_{{{\text{in}}}} }}{{{\text{dt}}}} = \sum\limits_{{{\text{i}} = {\text{1}}}}^{7} {{\text{h}}_{{{\text{in,i}}}} }  \times {\text{(T}}_{{{\text{bi}}}}  - {\text{T}}_{{{\text{in}}}} {\text{)}} \times {\text{A}}_{{\text{i}}}  + \rho _{{\text{a}}} {\text{c}}_{{{\text{pa}}}} {\text{V}}_{{\text{R}}} {\text{ACH(T}}_{{{\text{out}}}}  - {\text{T}}_{{{\text{in}}}} {\text{)/3600,}} $$

where ${\text{c}}_{\text{pa}}$ and ρ_a_ represent the indoor constant-pressure volumetric-specific heat and air density, respectively, ${\text{V}}_{\text{R}}$ is the building cubage,${\text{h}}_{\text{in,i}}$, ${\text{T}}_{\text{bi}}$, and ${\text{A}}_{\text{i}}$ denoted the convective thermal transfer coefficient, temperature, and $ith$ inner surface area, respectively, and ${\text{ACH}}$ is the ventilation air change rate.

The Gauss–Seidel technique is used in FORTRAN to numerically solve Eqs. (1), (2), (3), (4), (5), (6) and (7).

### Dynamic thermal performance of the buildings

#### Discomfort degree hours

The discomfort degree hours concept is proposed in a previous study^32^ and is expressed as follows:

The summer discomfort degree hours:

8 $$ {\text{I}}_{{{\text{sum}}}}  = \smallint _{0}^{{8760}} ({\text{T}}_{{{\text{in}}}}  - {\text{T}}_{{\text{H}}} )dt{\text{ when }}T_{{in}}  > T_{H} . $$

The winter discomfort degree hours:

9 $$ {\text{I}}_{{{\text{win}}}}  = \smallint _{0}^{{8760}} ({\text{T}}_{{\text{L}}}  - {\text{T}}_{{{\text{in}}}} )dt{\text{ when }}T_{{in}}  < T_{L} . $$

**Figure 3:**
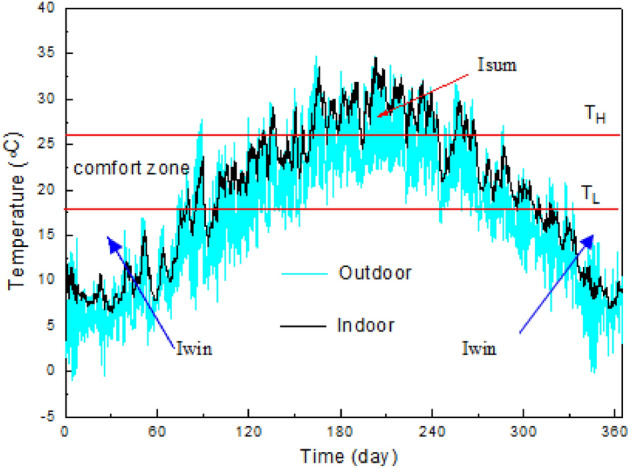
A schematic diagram of ${\text{I}}_{\text{sum}}$ and ${\text{I}}_{\text{win}}$.

The total annual discomfort degree hours can be expressed as follows:

10 $${\text{I}}_{\text{year}}={\text{I}}_{\text{sum}}+{\text{I}}_{\text{win}},$$

where ${\text{T}}_{\text{H}}$ and ${\text{T}}_{\text{L}}$ are the indoor higher-limit temperature for summer cooling and the lower-limit temperature for winter heating, respectively, here, T_H_ = 26 °C and T_L_ = 18 °C^33^. Figure 3 illustrates the indoor discomfort degree hours of ${\text{I}}_{\text{sum}}$ and ${\text{I}}_{\text{win}}$.

#### Building energy consumption

The yearly building model cooling load:

11 $$ {\text{Q}}_{{\text{C}}}  = \smallint _{{{\text{D}}_{{{\text{sum}}}} }} \sum\limits_{{{\text{i}} = {\text{1}}}}^{7} {{\text{h}}_{{{\text{in,i}}}} } \left( {{\text{T}}_{{{\text{bi}}}}  - {\text{T}}_{{\text{H}}} } \right) \times {\text{A}}_{{\text{i}}}  + \rho _{{\text{a}}} {\text{c}}_{{{\text{pa}}}} {\text{V}}_{{\text{R}}} {\text{ACH(T}}_{{{\text{out}}}}  - {\text{T}}_{{\text{H}}} )/3600. $$

The yearly building model heating load:

12 $$ {\text{Q}}_{{\text{H}}}  = \smallint _{{{\text{D}}_{{{\text{win}}}} }} \sum\limits_{{{\text{i}} = {\text{1}}}}^{7} {{\text{h}}_{{{\text{in,i}}}} } \left( {{\text{T}}_{{\text{L}}}  - {\text{T}}_{{{\text{bi}}}} } \right) \times {\text{A}}_{{\text{i}}}  + \rho _{{\text{a}}} {\text{c}}_{{{\text{pa}}}} {\text{V}}_{{\text{R}}} {\text{ACH(T}}_{{\text{L}}}  - {\text{T}}_{{{\text{out}}}} )/3600. $$

Therefore, the total yearly load can be expressed as follows:

13 $$\text{Q }={\text{ Q}}_{\text{C}}+{\text{Q}}_{\text{H}},$$

where ${\text{D}}_{\text{win}}$ and ${\text{D}}_{\text{sum}}$ represent the hours in the heating and cooling periods, respectively.

Since it is assumed that the cooling and heating equipment of the building include an air-source heat pump, the annual building heating energy consumption can be expressed as follows:

14 $$ {\text{C}}_{{{\text{A,H}}}}  = \frac{{{\text{Q}}_{{\text{H}}}  \cdot {\text{C}}_{{\text{E}}} }}{{{\text{3}}{\text{.6}} \times 10^{6}  \cdot {\text{COP}}_{{\text{H}}} }}, $$

where ${\text{C}}_{\text{E}}$ is the electricity price and ${\text{COP}}_{\text{H}}$ is the performance coefficient of the heating equipment, set to 1.9^33^.

Similarly, the annual cooling energy consumption of the building can be expressed as follows:

15 $${\text{C}}_{\text{A,C}}{=}\frac{{\text{Q}}_{\text{C}}{\cdot}{{\text{C}}}_{\text{E}}}{{3.6}\times{10}^{6}{\cdot}{{\text{COP}}}_{\text{C}}},$$

where ${\text{COP}}_{\text{C}}$ is the performance coefficient of the cooling equipment, set to 2.3^33^.

Therefore, the total annual building energy consumption is summarized as follows:

16 $$ {\text{C}}_{{\text{A}}}  = {\text{C}}_{{{\text{A,H}}}}  + {\text{C}}_{{{\text{A,C}}}} . $$

The total yearly building energy consumption in the presence of energy-saving measures is defined as ${\text{C}}_{\text{AE}}$. Then, the ESR can be expressed as follows:

17 $$ {\text{ESR}} = {\text{(1}} - {\text{C}}_{{{\text{AE}}}} /{\text{C}}_{{\text{A}}} {\text{)}} \times {\text{100}}{\text{.}} $$

### Calculation parameters

In this study, the outer boundary conditions were derived from the annual hourly meteorological data^34^ in Chengdu. The cool season spanned June 15 to August 15, while the hot period lasted from December 1 to March 15^35^. Table 4 presents the relevant parameters used for calculation.

**Table 4:** The calculation parameters.

Parameter	Value	Parameter	Value
${\alpha }_{win}$	0.12	${\alpha }$	0.56
${\varepsilon }_{win}$	0.12	${\varepsilon }$	0.56
${h}_{out,win}$	23 W/(m^2^⋅K)	${h}_{in,win}$	8 W/(m^2^⋅K)
${h}_{out}$(winter)	23.3 W/(m^2^⋅K)	${h}_{out}$(summer)	19 W/(m^2^⋅K)
${S}_{C}$	1.00	${h}_{in}$	8.7 W/(m^2^⋅K)

The natural ventilation strategy was described as follows: Natural ventilation was used in summer when the air temperature was lower outside than the comfortable higher-limit temperature ${T}_{H}$ inside. Consequently, the indoor and the outdoor air temperatures were the same. Natural ventilation was not considered in winter. The grid and time-step independence was determined using different grid sizes and time steps. Here, 0.5 mm and 10 s were selected as the grid size and time step, respectively.

### Optimization algorithm

Kheiri F.^36^ indicated that stochastic population-based algorithms, such as GAs, were most frequently used for building performance optimization. GAs are simple, robust, and suitable for searching the global optimum instead of a local one^37^. This study adopted an IGA^27^ as the search engine to determine the synergic relationships between the building design parameters and the energy-saving factors beneficial to energy-efficient building design. To maintain IGA operational efficiency during the search process, the study used 100 as the population size, 500 generations, a 0.96 crossover rate, and a 0.05 mutation rate. The procedural steps of this algorithm include the following:

*Step 1*: The target parameters were determined (i.e. the thermal building performance or energy efficiency indicators).

*Step 2*: The variable parameters were selected (e.g. the thermal property parameters of the envelope or the design parameters of the energy-saving measures). The variable parameters in this paper were differentiated according to the optimization objective and scenario. The parameters included the shading coefficient, thermal conductivity, and thickness of the insulation material.

*Step 3*: The variable parameters were encoded using the binary code, and ${\text{N}}$ individuals were randomly generated to constitute the initial population **P **(Gen = 1**)**.

*Step 4*: Each individual was decoded, which contained considerable parameter information in **P** (Gen). These parameters were entered into the building numerical calculation model (i.e. Eqs. (1), (2), (3), (4), (5), (6) and (7)) to determine the building thermal performance or the efficiency target of each individual (i.e. Formulas (8)–(17)).

*Step 5*: The indicators of each individual in **P** (Gen) were compared with the target parameters. The fitness value of each individual was evaluated.

*Step 6*: Step 7 was implemented when the termination condition was not satisfied, otherwise, step 12 was performed.

*Step 7*: The optimal individuals were selected from **P** (Gen) using the roulette selection method for correlation matching.

*Step 8*: The effective intersection zone for each parent generation was calculated, a multi-point crossover operation was performed, and new individuals were generated^27^.

*Step 9*: Multiple uniform mutation operations were performed to generate new individuals.

*Step 10*: A new population was generated and the parent population was updated by introducing a competition mechanism between the parents and offspring.

*Step 11*: When Gen = Gen + 1, step 4 was repeated.

*Step 12*: The calculation was terminated, and the corresponding variable and target parameters were produced.

**Figure 4:**
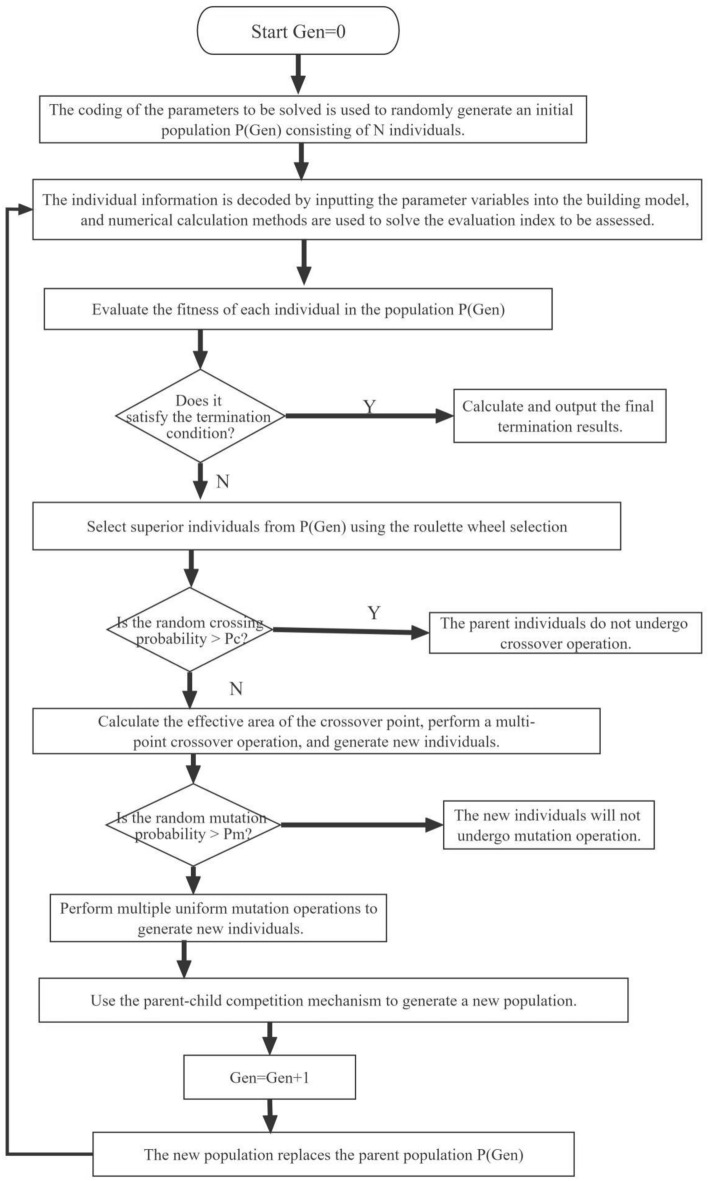
The procedural steps of this algorithm.

The procedural steps of this algorithm is shown in Fig. 4.

## Results and discussion

To find the best matching relationship between thermal property parameters, it is necessary to use the method of inverse problem, which is a means of studying the known effects to find their causes. In this section, an improved genetic algorithm coupled with a numerical calculation method is used to establish a mathematical model. Indoor comfort or annual energy consumption is taken as the constraint objective to seek the synergistic relationship between the thermal property parameters of the enclosure structure or energy-saving measures.

### Case study 1

The synergistic correlation between the thermophysical characteristics of the material used for insulation subject to ${\text{I}}_{\text{sum}}= {0}$.

In this case, the optimization objective was to ensure that the indoor air temperature dropped within the comfort zone in summer, i.e. ${\text{I}}_{\text{sum}}= {0}$. The synergic relationship between the volumetric-specific heat $(\rho {c}_{P})$, thermal conductivity $(\lambda$), and thickness $(\delta )$ of the insulation materials were investigated and could be described as follows:

Optimization objective: $${\text{I}}_{\text{sum}}= {0} $$

Constraint conditions:

18 $$\left\{\begin{array}{l}0\le \delta \le {L}_{max}\\ {\lambda }_{min}\le \lambda \le {\lambda }_{max}\\ {\rho c}_{P,min}\le \rho {c}_{P}\le {\rho c}_{P,max}\end{array}.\right.$$

Table 5 summarizes the boundary values of the thermal criteria.

**Table 5:** The variable boundary values in case study 1.

Variable	Min value	Max value
$\delta$ $\text{(m)}$	0	0.4
$\rho {c}_{P}$ (kJ/(m^3^⋅K))	30	650
λ (W/(m⋅K))	0.02	0.2

#### The synergistic correlation between the thermophysical properties and thickness of the insulation material under the combined action of natural ventilation and external insulation

**Figure 5:**
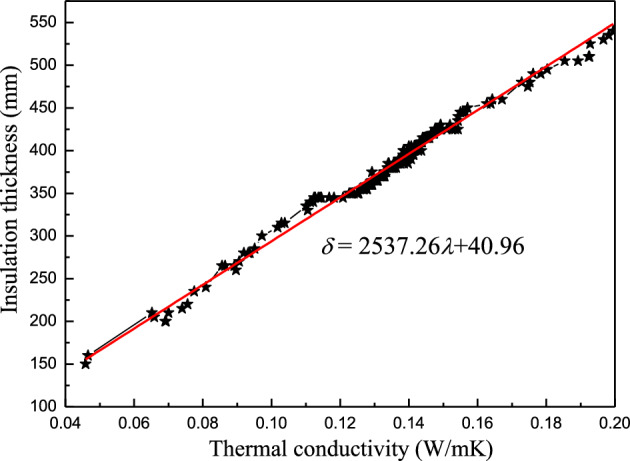
The synergistic correlation between the conductivity and thickness of the insulation material via natural ventilation and external insulation interaction.

In this section, the energy conservation measures are limited to natural ventilation and external insulation, while the shading technology is not considered, i.e. $\text{SC=1}$. The thickness of the thermal insulation refers to the critical thickness ensuring ${\text{I}}_{\text{sum}}=0$. Figure 5 shows the calculation results of the synergic relationship.

Figure 5 shows that the interaction between natural ventilation and external insulation meets the ${\text{I}}_{\text{sum}}= {0}$ stipulations. The indoor air temperature entirely satisfied the comfort requirements without air-conditioning if the design was reasonable. Therefore, the energy-saving effect was significant during the cooling period in Chengdu. Furthermore, the results revealed a linear synergic relationship between thermal conductivity and insulation thickness, which were proportional in ${\text{I}}_{\text{sum}}= {0}$. The linear fitting equation is obtained as follows:

19 $$\delta \text{= 2537.26}\lambda {+40.96.}$$

This relationship is extremely useful in practical engineering. To meet ${\text{I}}_{\text{sum}}= {0}$ in Chengdu, designers can select the appropriate thermal insulation material according to the local market conditions, while the critical thermal insulation thickness can be quickly calculated using Formula (19).

Analysis showed that $\rho {c}_{P}$ minimally affected the synergic thermal conductivity and insulation thickness relationship. As shown in Tables 2 and 4, the $\rho {c}_{P}$ of the insulation material was relatively low compared with the main body materials of the building. The main role of insulation material is increasing heat transfer resistance but not the thermal inertia of the envelope. Therefore, the effect of $\rho {c}_{P}$ on the synergistic correlation between the heat conductivity and insulation thickness can be ignored.

#### The synergistic correlation between the thermophysical properties and thickness of the insulation material under the combined action of natural ventilation, shading, and external insulation

**Figure 6:**
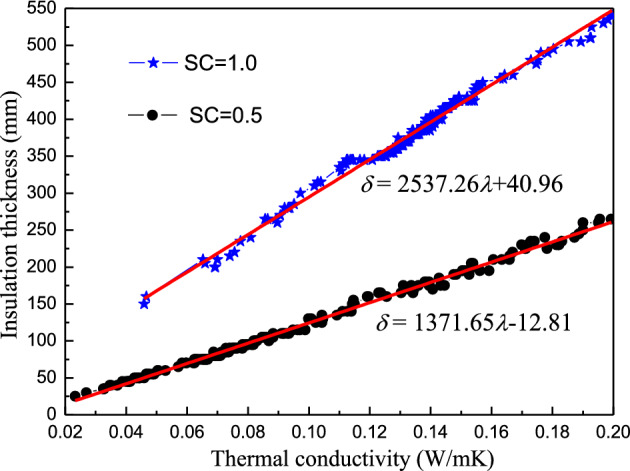
A comparison between the synergic conductivity and thickness relationships.

This section considers three energy-saving technologies simultaneously. The window shading coefficient was set as $\text{SC=0.5}$ in the calculation. The thermal insulation thickness represented the critical thickness that ensured ${\text{I}}_{\text{sum}}=0$. Figure 6 presents the calculated synergic relationships compared with those obtained in Sect.  “The synergistic correlation between the thermophysical properties and thickness of the insulation material under the combined action of natural ventilation and external insulation”.

As shown in Fig. 6, the synergic thermal conductivity and insulation thickness relationships were consistent regardless of whether shading technology was considered. The fitting equation, in this case, can be expressed as follows:

20 $$\delta { = 1371.65}\lambda {-12.81.}$$

A comparison between the two lines in Fig. 6 indicated that shading technology significantly impacted the synergistic correlation between the thermal conductivity and critical thickness of the material uses for insulation. The slope corresponding to $\text{SC=1}$ was larger, with a value of 2537.26, while that for $\text{SC=0.5}$ was smaller at 1371.65. However, the insulation thickness increment caused by the same thermal conductivity increment was larger without shading measures.

Furthermore, shading measures drastically reduced the critical thickness of the insulating material. Therefore, shading should be applied where possible in Chengdu since it decreases the initial investment in thermal insulation materials and reduces the discomfort degree hours during summer.

### Case study 2

The synergistic correlation between the thermal parameters of the insulation material subject to $\text{ESR=65}\%$.

China currently aims to save an average of 65% on building energy consumption. In this section, the energy conservation measures are limited to external insulation. Therefore, the problem can be expressed as follows:

Optimization objective: $${\text{ESR}}{=65\%}$$.

Constraint conditions:

21 $$\left\{\begin{array}{l}0\le \delta \le {L}_{max}\\ {\lambda }_{min}\le \lambda \le {\lambda }_{max}\\ {\rho c}_{P,min}\le \rho {c}_{P}\le {\rho c}_{P,max}\end{array}\right..$$

**Figure 7:**
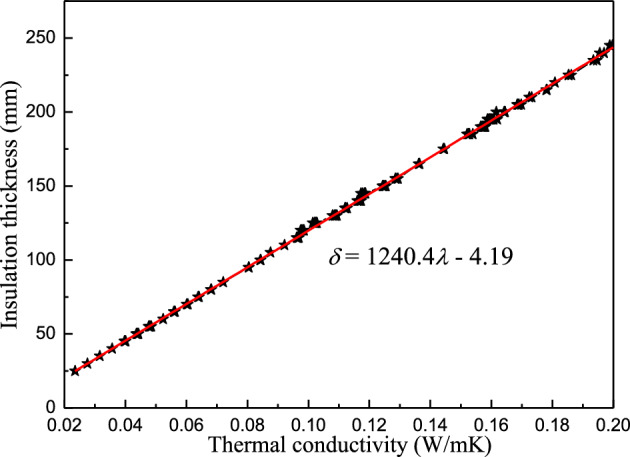
The synergistic correlation between the conductivity and thickness of the insulation material (external insulation, ${\text{ESR}}{=65\%}$).

The thermal parameter boundary values were the same as in Table 5. Figure 7 presents the calculation results in these conditions.

Since external insulation completely fulfilled the requirements for an ESR of 65% in Chengdu (Fig. 7), it should be implemented in new or existing buildings in this region. Furthermore, the synergistic correlation was linear, e.g. ${\text{ESR}}{=65\%}$, indicating that the heat conductivity and insulation thickness were proportional. The fitting equation is expressed as follows:

22 $$\delta { = 1240.4}\lambda {-4.19.}$$

The calculation simplicity is convenient for engineering applications. Analysis indicated that *ρc*_*P*_ minimally affected the synergistic correlation between the insulation thickness and heat conductivity. Moreover, the plane was separated into two regions by the synergic relationship line (${\text{ESR}}{=65\%}$) (Fig. 7), namely the lower ${\text{ESR}}$ area (${\text{ESR}}{<65\%}$) on the bottom right and the higher ${\text{ESR}}$ area (${\text{ESR}}{>65\%}$) on the top left. Designers can select appropriate parameters to meet energy-saving requirements. As shown in Figs. 5, 6, and 7, the synergistic correlation predicted by the Formulas (19), (20), and (22)) allowed the selection of suitable, accurate insulation materials and thicknesses that fulfill engineering requirements.

### Case study 3

The synergic relationship between the parameters of the energy-saving measures subject to ${\text{I}}_{\text{sum}}= {0}$.

In this case, the energy-saving measures are limited to external insulation and shading technology, while the optimization objective is ${\text{I}}_{\text{sum}}= {0}$. The synergic insulation thickness (*δ*) and shading coefficient $({\text{S}}_{\text{c}}$) relationship is explored. This problem can be described as follows:

Optimization objective: $${\text{I}}_{\text{sum}}= {0} $$.

Constraint conditions:

23 $$\left\{\begin{array}{l}{0}\le \delta \le {\delta }_{\text{max}}\\ {{\text{S}}_{\text{c}}}_{\text{min}}\le {\text{S}}_{\text{c}}\le {{\text{S}}_{\text{c}}}_{\text{max}}\end{array}.\right.$$

Table 6 summarizes the parameter boundary values.

**Table 6:** The variable boundary values in case study 3.

Variable	Min value	Max value
$\delta$ $\text{(m)}$	0	0.45
${\text{S}}_{\text{c}}$	0.01	1

#### The synergistic correlation between the shading coefficient and insulation thickness

**Figure 8:**
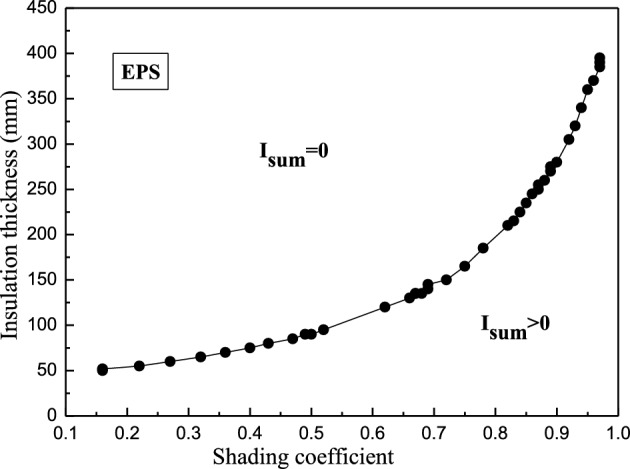
The synergistic correlation between the thickness of EPS and the shading coefficient.

Due to its popularity in China, EPS (expanded polystyrene) is used as the insulation material in this section. Figure 8 shows the synergistic correlation between the thickness of EPS and the shading coefficient.

An exponential relationship was evident between the EPS thickness and shading coefficient (Fig. 8). The insulation thickness increased at a higher shading coefficient. The initially gentle curve became steep when the shading coefficient reached around 0.6. Therefore, a lower shading coefficient after this point caused a more significant insulation thickness increase. In addition, the plane was separated into two regions by the synergic relationship curve (Fig. 8), namely the bottom-right ${\text{I}}_{\text{sum}}> {0}$ area and the upper-left ${\text{I}}_{\text{sum}}= {0}$ area, while the curve was critical. In practical engineering, the designer can obtain the critical EPS thickness for any shading coefficient based on this curve. Furthermore, the additional insulation thickness did not reduce the indoor discomfort degree hours in summer.

#### The effect of *ρc*_*P*_ on synergic shading coefficient and insulation thickness relationship

**Figure 9:**
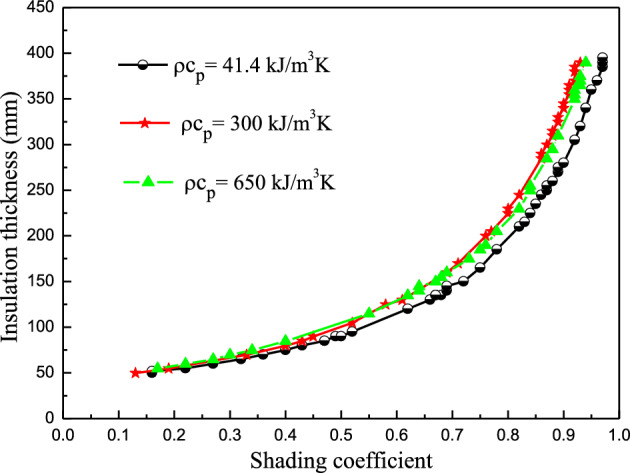
The effect of *ρc*_*P*_ on the synergic shading coefficient and insulation thickness relationship.

In this section, it is assumed that the thermal conductivity of the insulation material is fixed as the EPS value, while the *ρc*_*P*_ of the insulation material is set as 41.4 kJ/(m^3^∙K), 300 kJ/(m^3^∙K), and 650 kJ/(m^3^∙K), respectively. Figure 9 shows the corresponding synergic relationships at the three *ρc*_*P*_ values.

The different volumetric heat capacity curves showed similar trends. At a shading coefficient below 0.35, the three curves coincided almost exactly. At a shading coefficient higher than 0.35, the curves of *ρc*_*P*_ = 300 kJ/(m^3^∙K) and *ρc*_*P*_ = 650 kJ/(m^3^∙K) almost coincided and were located above that of *ρc*_*P*_ = 41.4 kJ/(m^3^∙K). Therefore, the volumetric heat capacity did not significantly impact the insulation thickness when the shading coefficient was smaller (less than 0.35). However, the impact could not be ignored when the shading coefficient was larger (above 0.35).

#### The impact of the insulation material heat conductivity on the synergic relationship between the shading coefficient and insulation thickness

**Figure 10:**
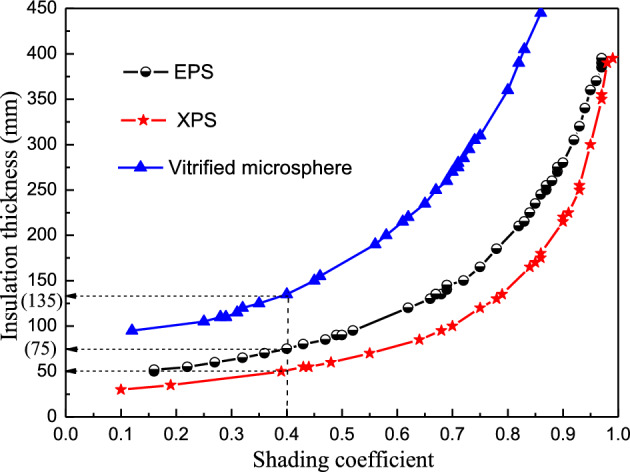
The effect of conductivity on the synergic insulation thickness and shading coefficient relationship.

Three popular insulation materials, namely EPS, XPS, and a vitrified microsphere, were compared. Table 7 summarizes the thermophysical properties of these three materials. Figure 10 plots the impact of heat conductivity on the synergic relationship between the shading coefficient and insulation thickness.

**Table 7:** The thermophysical characterisics of the three insulation materials.

Material	*λ* (W/m ⋅ K)	*ρ* (kg/m^3^)	${c}_{p}$(J/kg ⋅ K)
XPS	0.028	35	1380
EPS	0.042	30	1380
Vitrified microsphere	0.069	270	1050

The synergic relationships between the shading coefficients and thicknesses of the three materials used for insulation displayed similar exponential trends (Fig. 10), while the thermal conductivity played a decisive role. The vitrified microsphere curve displayed the highest thermal conductivity, while the XPS curve exhibited the lowest. The critical insulation thickness for any shading coefficient of each material can be achieved by the curve. For example, at a shading coefficient of 0.4, the critical insulation thicknesses were 50 mm for XPS, 75 mm for EPS, and 135 mm for the vitrified microsphere, respectively. Therefore, the critical insulation thickness increased at a higher thermal conductivity for the fixed shading coefficient.

### Case study 4

The synergistic correlation between the thermal parameters of the energy-saving measures subject to $\text{ESR=65}\%$.

In this case, the optimization objective is ${\text{ESR}}{=65\%}$. The synergistic correlation between the thickness (*δ*) and shading coefficient $({\text{S}}_{\text{c}}$) of the insulation material was investigated. This problem can be described as follows:

Optimization objective: $${\text{ESR}}{=65\%}$$.

Constraint conditions:

24 $$\left\{\begin{array}{l}{0}\le \delta \le {\delta }_{\text{max}}\\ {{\text{S}}_{\text{c}}}_{\text{min}}\le {\text{S}}_{\text{c}}\le {{\text{S}}_{\text{c}}}_{\text{max}}\end{array}.\right.$$

#### The synergic insulation thickness and shading coefficient relationship for EPS

**Figure 11:**
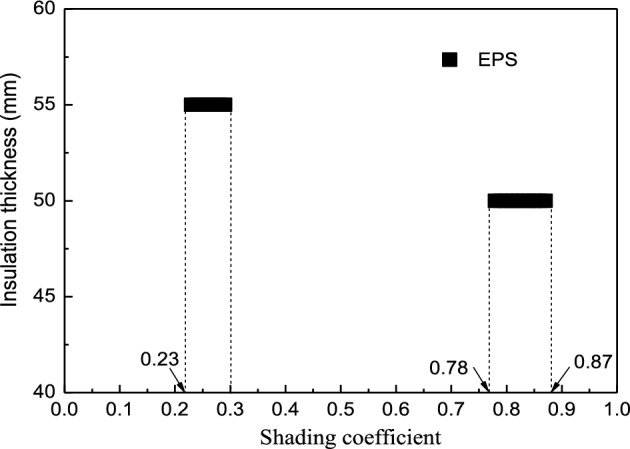
The synergistic correlation between the insulation thickness and shading coefficient of EPS.

The insulation material was limited to EPS. The parameter boundary values were the same as in Table 6. Figure 11 shows the synergistic correlation between the shading coefficient and insulation thickness of EPS.

The 65% ESR can be achieved in Chengdu via external insulation and shading technology synergy (Fig. 11). The synergistic correlation between the EPS shading coefficient and insulation thickness was displayed as a segmented function. To meet the 65% ESR requirements of the building, suitable thicknesses of 50 mm and 55 mm were used. At a fixed EPS thickness of 50 mm, the synergic shading coefficient ranged between 0.78 and 0.87 and from 0.23 to 0.3 at 55 mm. Therefore, the conclusion can be drawn that to reach the same energy-saving effect, the smaller shading coefficient is matched with the larger insulation thickness. Further analysis showed that at a 50-mm EPS thickness, the energy-saving was below 65% when the shading coefficient was lower than 0.78, while it exceeded 65% at a shading coefficient over 0.87.

In addition, the contribution of the external insulation and shading technology to the ESR varied, that is, the external insulation was dominant while the shading technology was subordinate (Fig. 11). Therefore, thermal insulation technology should be considered as an energy-saving measure in Chengdu.

#### The synergic relationships between the shading coefficients and insulation thicknesses of the different materials

**Figure 12:**
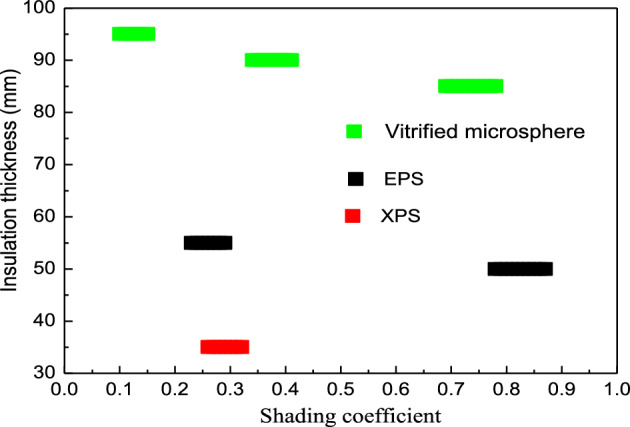
The synergic relationships between the shading coefficients and insulation thicknesses of the different insulation materials.

This section used EPS, XPS, and a vitrified microsphere as the insulation materials, while the synergic relationships for the different insulation materials were plotted (Fig. 12).

Figure 12 shows that the synergic relationships between the insulation thicknesses and shading coefficients of the three materials all presented segmented functions. To reach the same ESR of 65%, the synergic thickness of XPS was the smallest due to its lowest thermal conductivity. Only the 0.23 to 0.32 shading coefficient segment corresponded to the 35-mm EPS thickness. The combinable modes for the vitrified microsphere were diverse since they displayed the most significant thermal conductivity. The corresponding shading coefficients for insulation thicknesses of 85 mm, 90 mm, and 95 mm were 0.69 to 0.78, 0.34 to 0.41, and 0.1 to 0.15, respectively.

Besides, the synergic relationships indicate that to get the same ESR, the corresponding insulation thickness increases with the increase of the thermal conductivity. Furthermore, the insulation material thickness decreased when the synergic shading coefficient increased. The decline was reflected by the lower value and the short interval length (Fig. 12).

## Conclusions

To design energy-saving buildings that meet the comfort requirements specified in standards or norms, it is necessary to examine the synergistic correlation between thermophysical construction material characteristics and energy-saving technology parameters. This paper uses combines IGA and numerical calculations to examine the synergic relationships feasible for engineering applications. The study concentrates on a single-zone building in the diverse climate of Chengdu, China. The primary findings can be summarized as follows:

1. Considering external insulation and natural ventilation, the insulation material thickness and heat conductivity are linearly associated for ${\text{I}}_{\text{sum}}= {0}$. The insulation thicknesses are proportional to the thermal conductivities, while the synergic relationships are almost independent of $\rho {c}_{P}$. When shading technology is considered synchronously, the shading coefficient significantly influences the linear synergic insulation thickness and thermal conductivity relationships.
2. When assuming the external insulation and shading technology in ${\text{I}}_{\text{sum}}= {0}$, the thickness and shading coefficient are exponentially related. The thermal conductivity of the insulation material significantly affects the synergic relationship. The synergic relationship curve of the vitrified microsphere is at the top because of higher thermal conductivity, while that of XPS is at the bottom due to lower thermal conductivity.
3. When assuming the external insulation and shading technology, a segmented function is evident in the insulation thickness and shading coefficient relationship for ${\text{ESR}}{=65\%}$. The thermal conductivity of the insulation material has a decisive influence on the synergic relationship. The synergic relationship displays more segments at a higher thermal conductivity. The insulation material thickness decreases when the synergic shading coefficient increases. This decline is reflected by the lower value and short interval.
4. Analyzing four cases confirms the veracity and reliability of the proposed technique for assessing the synergistic correlation between energy-saving technology parameters and thermophysical building material properties. The synergic relationships revealed in this study can significantly benefit practical engineering. This simplifies the selection of energy-saving technology combinations to satisfy local market conditions and design requirements.
5. This study only analyzes a typical building in hot summer and cold winter regions, and the research object is relatively simple, with fewer optimization parameter variables involved. Numerical calculations are based on a one-dimensional unsteady heat transfer model. In the future work, different climatic zones and more complex building forms can be considered, and the synergistic relationship between design parameters can be studied based on 2D or even 3D heat transfer processes to provide more specific and feasible directions for energy-efficient design.

## Data Availability

No datasets were generated or analysed during the current study.
